# Mechanisms of Mechanical Force Induced Pulmonary Vascular Endothelial Hyperpermeability

**DOI:** 10.3389/fphys.2021.714064

**Published:** 2021-09-04

**Authors:** Yan Lai, Yongbo Huang

**Affiliations:** ^1^State Key Laboratory of Respiratory Diseases, Guangzhou Institute of Respiratory Health, First Affiliated Hospital of Guangzhou Medical University, Guangzhou, China; ^2^Department of Critical Care Medicine, First Affiliated Hospital of Guangzhou Medical University, Guangzhou, China

**Keywords:** mechanical ventilation, pulmonary vascular endothelium, lung injury, permeability, mechanisms

## Abstract

Mechanical ventilation is a supportive therapy for patients with acute respiratory distress syndrome (ARDS). However, it also inevitably produces or aggravates the original lung injury with pathophysiological changes of pulmonary edema caused by increased permeability of alveolar capillaries which composed of microvascular endothelium, alveolar epithelium, and basement membrane. Vascular endothelium forms a semi-selective barrier to regulate body fluid balance. Mechanical ventilation in critically ill patients produces a mechanical force on lung vascular endothelium when the endothelial barrier was destructed. This review aims to provide a comprehensive overview of molecular and signaling mechanisms underlying the endothelial barrier permeability in ventilator-induced lung jury (VILI).

## Introduction

Acute respiratory distress syndrome (ARDS) is a condition in which lungs sustain an acute, diffuse, and inflammatory injury (Force et al., 2012; Fan et al., 2018). In an international, multicenter, prospective cohort study of patients admitted to ICUs, 10.4% of patients fulfilled ARDS criteria, and 23.4% require mechanical ventilation (Bellani et al., 2016). Mechanical ventilation is a supportive therapy for patients with ARDS; a lower tidal volume ventilation decreased mortality from 39.8 to 31% and increased the number of days without ventilator use (Brower et al., 2000). However, mechanical ventilation inevitably damages or aggravates the original lung injury characterized by inflammatory-cell infiltrates, formation of hyaline membranes, increased vascular permeability, and pulmonary edema (Slutsky and Ranieri, 2013). Increased permeability of alveolar capillaries enables fluid to leak into the interstitium and alveoli, resulting in clinical respiratory failure (Beasley, 2010; Murray, 2011). Exploring the mechanisms of endothelial layer responding to mechanical force is vital to develop effective endothelial-targeted treatments among patients who require mechanical ventilation. The primary purposes of this review are to (1) illustrate the increase of pulmonary microvascular endothelial permeability induced by mechanical ventilation, (2) summarize the destruction of endothelial barrier function caused by mechanical force, and 3) describe the molecular mechanisms involved in endothelial barrier disfunction.

## Increased of Microvascular Permeability Caused by Mechanical Ventilation

Under physiological conditions, the alveolar capillaries act as the first defense line, restricting fluid and macromolecules from entering the interstitium from blood (Parthasarathi, 2018). Fluid leaks across the vascular endothelium and accumulates in the interstitial space and alveoli, causing cardiogenic and non-cardiogenic edema (Murray, 2011). In 1974, Webb and Tierney first used a rat model and proved that high tidal volume ventilation (HTV) can cause fulminant non-cardiogenic edema (Webb and Tierney, 1974), consistently associated with alterations in pulmonary vascular permeability barrier (Dreyfuss and Saumon, 1993). Egan and Parker found that capillary filtration coefficient was immediately increased when airway pressures exceeded 30cm H_2_O threshold, and lung lymph protein clearances and minimal lymph or plasma ratios of total protein were significantly higher in high peak airway pressures group than in low peak airway pressures group, indicating a significant increase in microvascular permeability (Egan, 1982; Parker et al., 1990). Later, Katira et al. reproduced the data reported by Webb and Tierney that pulmonary edema is due to a large increase in microvascular permeability after mechanical ventilation (Katira et al., 2017). In critically ill patients with mechanical ventilation, decreased plasma albumin and transferrin levels parallel increased pulmonary vascular permeability, regardless of the underlying disease or fluid status (Aman et al., 2011).

## Dysfunction of Pulmonary Microvascular Endothelial Barrier

The destruction of lung gas-blood barrier function is characterized by significant changes in endothelial cell (EC) function (Birukova et al., 2006). ECs are located on the inner side of blood vessels, forming a selective semi-permeable barrier regulating body fluid balance and migration of white blood cells (Mehta and Malik, 2006). Mechanical ventilation induced excessive stretch of alveoli that transmits pathological mechanical stress to alveolar epithelium and pulmonary endothelium. *In vitro* studies through cyclic stretch (CS) epithelial cells indicate that 25% alveolar epithelial cell basal surface area increase is equated *to* exposure to 5% CS, reflects the physiological levels of mechanical stress in alveolar epithelium, and 40–50% alveolar epithelial cell basal surface area increase is equated *to* exposure to 18% CS, reflects high tidal volume mechanical stress in alveolar epithelium (Tschumperlin and Margulies, 1999; Wirtz and Dobbs, 2000). There are *no* studies report the association between capillary endothelium basal surface and ventilation, but *some scholars* consider *that* lung inflation transmits stress to capillary endothelium in a similar extent. Dreyfuss et al. found that an ultrastructural injury of ECs after mechanical ventilation occurs when the thin part of an EC is detached from the basement membrane and floats in the capillary lumen (Dreyfuss et al., 1985; Dreyfuss and Saumon, 1998). Structural and mechanical studies indicate that loss of ECs integrity induces endothelial permeability increase (Wang et al., 2012a). The endothelial permeability is mainly controlled by paracellular and transcellular permeability.

### Paracellular Permeability

Interactions between cells are essential for maintaining a tight endothelial barrier. Inter-endothelial junctions including adhesion junctions (AJs), gap junctions (GJs), tight junctions (TJs), and adhesion molecules (AMs) jointly maintain paracellular permeability balance and control the passage of plasma proteins, solutes, and fluid across the endothelial barrier (Komarova and Malik, 2010). Mechanical stress associated with VILI significantly impacts the expression of EC junctions, which leads to injurious remodeling and ECs barrier disruption.

### Adhesion Junctions

Adhesion junctions (AJs) are composed of vascular endothelial (VE)-cadherin complexes with catenins. VE-cadherin is an exclusive endothelium signature and the main component of endothelial AJs, comprising five extracellular cadherin domains, a transmembrane domain, and a highly conserved cytoplasmic tail, mediating intercellular adhesions of ECs through cis- and trans-dimerization (Rho et al., 2017). The cytoplasmic tail of VE-cadherin is combined with p120-catenin, β-catenin and plakoglobin. The latter two are connected to the actin cytoskeleton through the bridging effect of α-catenin (Dejana et al., 2008). VE-cadherin mediated the mechanical force transduction signals that increased integrin-dependent cell contractility and disrupted cell–cell and cell–matrix adhesions (Andresen Eguiluz et al., 2017). The expression of VE-cadherin is closely related to intercellular adhesions and ECs barrier function (Dejana and Vestweber, 2013). In tightly connected cells, phosphorylated VE-cadherin is at a low expression level, indicating a negative correlation between the integrity of AJs and phosphorylated VE-cadherin (Chiu et al., 2004). HTV combined with hyperoxia further activated Src to phosphorylate VE-cadherin at Y685 and Y658, leading to internalization and ubiquitination of VE-cadherin (Li et al., 2015; Soni et al., 2017; Zhong et al., 2019). The transmembrane domain of VE-cadherin mediates an essential adapter function by binding directly to the transmembrane domain of vascular endothelial growth factor receptor 2 (VEGFR2), thereby assembling the endothelial mechanosensory complex (Coon et al., 2015). Eighteen percent CS induced the time-dependent upregulation of VEGFR2 and dissociating VEGFR2 from VE-cadherin at cell junctions, which result in VEGFR2 activation, Src-dependent phosphorylation of VE-cadherin at Tyr658 and internalization. However, the disruption of AJs integrity was attenuated with VEGFR2 knockdown (Gawlak et al., 2016; Tian et al., 2016). The stability of AJs maintained by Yes-associated protein, and deletion of Yes-associated protein in ECs exaggerated VE-cadherin phosphorylation, downregulation of vascular endothelial protein tyrosine phosphatase, and dissociation of VE-cadherin and catenins following mechanical ventilation (Su et al., 2021). Deubiquitinating enzymeubiquitin carboxyl terminal hydrolase 1 is a protective factor in VILI of increased lung vascular permeability, as inhibition of ubiquitin carboxyl terminal hydrolase 1 was associated with decreased expression levels of VE-cadherin (Mitra et al., 2021).

### Gap Junctions

Gap junctions (GJs) are mainly composed of connexins (Cx); 4–6 connexins assembled in the plasma membrane to form a connexon, allowing ions and small signaling molecules to diffuse freely between neighboring cells (Yeager and Harris, 2007; Wang et al., 2017a). In ECs, GJs play an important role in regulating the permeability of the endothelial monolayer. Cx37, Cx40, and Cx43 are abundantly expressed in different lung ECs and involved in endothelial permeability (Nagasawa et al., 2006; Hautefort et al., 2019). Cx37 functions as a break of ECs proliferation and overexpression of Cx37 facilitates ECs apoptosis, indicating that Cx37 plays a crucial role in angiogenesis and vascular repair (Seul et al., 2004; Pohl, 2020). However, there are no reports on the specific role of Cx37 in endothelial repair after mechanical ventilation or mechanical stretch. Cx40 induces Rho associated coiled-coil containing protein kinase 1 (ROCK1) upregulation and subsequent phosphorylation of myosin phosphatase target subunit and myosin light chain 20 (MLC20). Inhibition of Cx40 suppresses cells proliferation; however, genetic deficiency of Cx40 significantly attenuated inflammation, lung edema, and histological evidence of lung damage (Yin et al., 2019). Cx43 regulates lung endothelial barrier function by increasing phosphorylation of MLC and ROCK1 expression, affecting cell contraction and stress fiber formation (Zhang et al., 2015). Immunofluorescence and immunoblot analyses revealed that both Cx43 expression and micro-vessel permeability increased above baseline within a few hours after endotoxin instillation but declined progressively over the next few days, and the knockdown of vascular Cx43 by Cx43 shRNA increased VE-cadherin expression, suggesting that the reduction in Cx43 levels may modulate VE-cadherin levels in lung micro-vessels. In contrast to Cx43, the expression of micro-vessel VE-cadherin exhibited an inverse trend by initially declining below baseline and then returning to baseline at a longer duration (Kandasamy et al., 2015). Eighteen percent CS causes a significant increase of Cx43 at both protein and mRNA levels, and the GJs inhibitor, carbenoxolone, attenuates the permeability response to thrombin and inhibits MLC phosphorylation, Cx43 siRNA transfection significantly increased cell viability, inhibited cells apoptosis and caspase-3 activity (Cowan et al., 1998; O'Donnell 3rd et al., 2014). Cx37 or Cx40 seems to be less mechanosensitive, but Cx43 is mechanosensitive and plays an essential role in CS induced EC barrier regulation and possibly be a novel therapeutic target (Salameh and Dhein, 2013).

### Tight Junctions

Tight junctions (TJs) are intramembrane multiprotein complexes composed of transmembrane proteins, including occludins, claudins, junction adhesion molecules, and cytoplasmic scaffold protein zonula occludins (ZO) that create a paracellular barrier in epithelial and endothelial cells and protect epithelial and endothelial cell from the external environment (Forster, 2008; Hartsock and Nelson, 2008; Cummins, 2012). Similar to AJs, the TJs is affected by VILI associated stress.

### Claudins

The claudins family contains at least 24 members whose expression is tissue-specific; ECs specifically express claudin-5 (Forster, 2008). Studies established claudin-5 as being key for TJs formation in ECs. Claudin-5 gene knockout mice are selectively permeable to small molecules (Morita et al., 1999). At the transcriptional level, claudin-5 is regulated by the transcription factor Sox-18, which plays a key role in promoting blood vessel development and maintaining stability of endothelial barrier function (Fontijn et al., 2008). The shear stress up-regulates Sox-18 expression, and in turn, increases claudin-5 expression, maintains the stability of endothelial barrier function and prevents cardiogenic pulmonary edema (Gross et al., 2014). In contrast, LPS plus mechanical ventilation dramatically reduced claudin-5 expression in lung tissues, further destroyed TJs structure, and increased endothelial permeability (Li et al., 2020). Oxycodone is a synthetic opioid receptor agonist that decreased pulmonary microvascular permeability both *in vivo* and *in vitro* by upregulating the expression of claudin-5 (Li et al., 2021). The regulation of claudin-5 in the vascular endothelium can greatly impact TJs formation and endothelial barrier function (Wang et al., 2017a; Kakogiannos et al., 2020). However, our knowledge regarding the role of claudin-5 in mechanical force induced lung ECs barrier dysfunction has not been fully studied.

### Occludin

Occludin is tetrameric protein that express in lung epithelial cells and ECs, and the N-terminus corresponds to several properties of the TJs barrier (Furuse et al., 1993; Zhang and Yang, 2016). The phosphorylation states of occludin affect its localization: non-phosphorylated occludin is localized at both the basolateral membrane and in cytoplasmic vesicles, and phosphorylated occludin is localized at TJs. The regulation of barrier function based on occludin in endothelial cells was correlated with its phosphorylation and expression level (Zhang and Yang, 2016). Mechanical ventilation activated PKC signaling pathway and decreased occludin expression in a HTV group than a LTV group. Pretreatment with a PKC inhibitor increased occludin expression and could reduce or delay pulmonary edema (Liu et al., 2014). Adaptive support ventilation is a closed-loop ventilation, which upregulated expression level of occludin and exhibited less lung injury and greater alveolar fluid clearance compared with the volume-control ventilation group (Dai et al., 2019b). Mechanical ventilation activated c-Src by phosphorylation and decreased occludin expression, while c-Src inhibitor upregulated occluding expression, which ameliorates barrier function (Zhao et al., 2014).

### Adhesion Molecules

Adhesion molecules (AMs) include integrins and immunoglobulin superfamily cell adhesion molecules (IgCAMs), allowing the endothelium to form attachments with surrounding proteins and cells to modify endothelial barrier function and vascular permeability (Sarelius and Glading, 2015).

### Integrins

Integrins are heterodimer transmembrane proteins that consist of two subunits, containing extracellular, transmembrane, and cytoplasmic domains, 18 α subunits and 8 β subunits that can assemble into 24 different receptors, which has different binding properties and different tissue distribution (Campbell and Humphries, 2011). Integrins are known to mediate vascular stability by mediating ECs adhesion to vascular basement membrane (Yamamoto et al., 2015). In ECs, integrin β1 dependent ECs basement membrane adhesions contribute to acute endothelial permeability responses, heterozygous deletion of integrin β1 protected the mice from LPS-induced vascular leakage (Hakanpaa et al., 2018). Integrins serve as bidirectional hubs transmitting signals between cells and their environment and respond to extracellular matrix, which mediates the formation of mechanosensitive structures (Kechagia et al., 2019; Sun et al., 2019a). Integrins can be activated by mechanical force and involved in vascular permeability regulation (Sarelius and Glading, 2015; Gingras and Ginsberg, 2020). Integrins β5 is specifically expressed in the pulmonary endothelium and involved in VILI. Integrin αvβ5 contributes to stress fiber formation and increases endothelial permeability by activating downstream of RhoA. Function-blocking antibody against integrin αvβ5 prevented lung vascular permeability development in VILI mice, and the same effects were also found in integrin αvβ5 gene knockout mice (Su et al., 2007). Cecal ligation and puncture (CLP) led to small but significant increases in integrin β5. However, HTV combined with CLP group increased integrin β5 expression 2–3 folds more than CLP group, and anti-integrin β5 antibodies partially inhibited the two-hit phenotype of increases in alveolar-capillary permeability, histopathologic scoring, and indices of pulmonary inflammation in mice (Ding et al., 2018). Moderate tidal volume mechanical ventilation further increased poly(I:C), an analog of natural double-strand RNA virus induced integrin β3 expression in the lung, inhibited integrin ανβ3, reduced inflammation and attenuated VILI in rats (Wang et al., 2012b; Jin et al., 2016). Mechanical ventilation or CS induces the phosphorylation of integrins at different positions (Kelly et al., 2019). Integrins activated within 1min after exposing to 15% CS, as indicated by increased phosphorylation of the T788/789 site of the integrin cytoplasmic tail (Thodeti et al., 2009). Tyrosine phosphorylation of integrin β4 at Y1440, Y1526, Y1640, or Y1422 in response to 18% CS was evident within 30min and peaked at 2h, and integrin β4 mutant mice demonstrated decreased lung vascular permeability and inflammation when subjected to mechanical ventilation (Chen et al., 2015). For other integrins, integrin β8 has a non-classical cytoplasmic domain and plays a role in angiogenesis integrin β6 is principally expressed in epithelium and play an important role in septic mice by regulating lung inflammatory and alveolar capillary permeability, integrins β2 and β7 are principally expressed in leukocytes and involve in inflammation (Tabata et al., 2008; Giusti et al., 2013; Chen et al., 2015; Ding et al., 2015, 2018; Ou et al., 2021). The involvement of integrins in mechanical force induced endothelial permeability is limited and requires further experiments.

### IgCAMs

Platelet-endothelial cell adhesion molecule 1 (PECAM-1), a member of IgCAMs family, is located at ECs intercellular junctions, where it functions as a mechanosensor and maintenance of ECs junctional integrity (Privratsky and Newman, 2014; Liao et al., 2018). As an efficient signaling molecule, PECAM-1 has diverse vascular biology roles, including angiogenesis, platelet function, thrombosis, mechanosensing of fluid shear stress, and regulating leukocyte migration (Woodfin et al., 2007). Five percent CS alone did not cause any significant changes in cell area and cell aspect ratio compared to untreated control cells, but cell area increased significantly in response to concurrent shear stress and CS (Meza et al., 2019). Upon CS, the cytoplasmic domain of PECAM-1 is unfolded and exposes a phosphorylation site, allowing the rapid PECAM-1 phosphorylation, transforming a mechanical signal into a biochemical signal (Snyder et al., 2017). Five percent CS caused 42% increase in PECAM-1 phosphorylation (Meza et al., 2019). In a rat VILI model, mechanical ventilation decreased PECAM-1 expression in the lung, released soluble sPECAM1 into the blood circulation, and caused damage to the pulmonary permeability barrier as showed by pulmonary edema, leukocyte infiltration, and severe hypoxemia (Villar et al., 2014).

### Transcellular Permeability

Transcellular permeability *via* transcellular vesicle trafficking selectively transports macromolecules such as albumin and albumin-bound ligands, insulin, lipids, and hormones from vessel lumen to interstitial space (Komarova and Malik, 2010). Caveolae, flask-shaped non-coated structures, compose 20% of cell volume in ECs and mediate transcytosis to regulate endothelial transcellular permeability (Komarova and Malik, 2010). Caveolae contain a membrane protein known as caveolin (Cav), including caveolin-1, −2, and−3. ECs express high levels of Cav-1 involved in endothelial inflammation, adhesion, and phagocytosis (Shihata et al., 2016).

Cav-1 is a structural protein of caveolae with N and C-terminal cytoplasmic tail with function domains: tyrosine phosphorylation site and oligomerization domain can regulate the endothelial transcellular permeability (Sun et al., 2011; Boscher and Nabi, 2012). To explore the role of Cav-1 in regulating lung vascular permeability, Miyawaki-Shimizu et al. used small interfering RNA to knock down Cav-1 expression in mouse lung endothelia *in vivo*. Caveolae disappearance in lung vessel endothelia is concomitant with increase lung vascular permeability to albumin (Miyawaki-Shimizu et al., 2006). The function of Cav-1 is regulated by Src-mediated phosphorylation of Cav-1 at Tyr14 initiating plasmalemmal vesicle fission and transendothelial vesicular transport (Sun et al., 2009; Boscher and Nabi, 2012). Src was phosphorylated and accompanied by synchronous phosphorylation of Cav-1 at Tyr14 after 30min of mechanical ventilation with a tidal volume of 21ml/kg on mice (Maniatis et al., 2012). Cav-1^−/−^ mice show less susceptibility to pulmonary endothelial hyperpermeability induced by HTV, but rescue of Cav-1 expression restored the hyperpermeability response to HTV, indicating that caveolin-1 expression is required for mechanical force-induced endothelial hyperpermeability (Maniatis et al., 2012; Zhong et al., 2015). In contrast, Hoetzel found that ventilation with 12ml/kg for 8h increased Cav-1 expression levels, and Cav-1 deficiency aggravated lung injury characterized by protein leakage, edema formation, and macrophage infiltration, suggesting that Cav-1^−/−^ mice were more susceptible to pulmonary endothelial hyperpermeability induced by mechanical ventilation (Hoetzel et al., 2009). The different results may depend on mechanical ventilation parameters. Cav-1 expression in transcellular permeability is controversial, and more research is needed to explore the role of Cav-1 in mechanical force-induced endothelial hyperpermeability.

## Molecular Mechanisms in Endothelial Permeability

**Figure 1:**
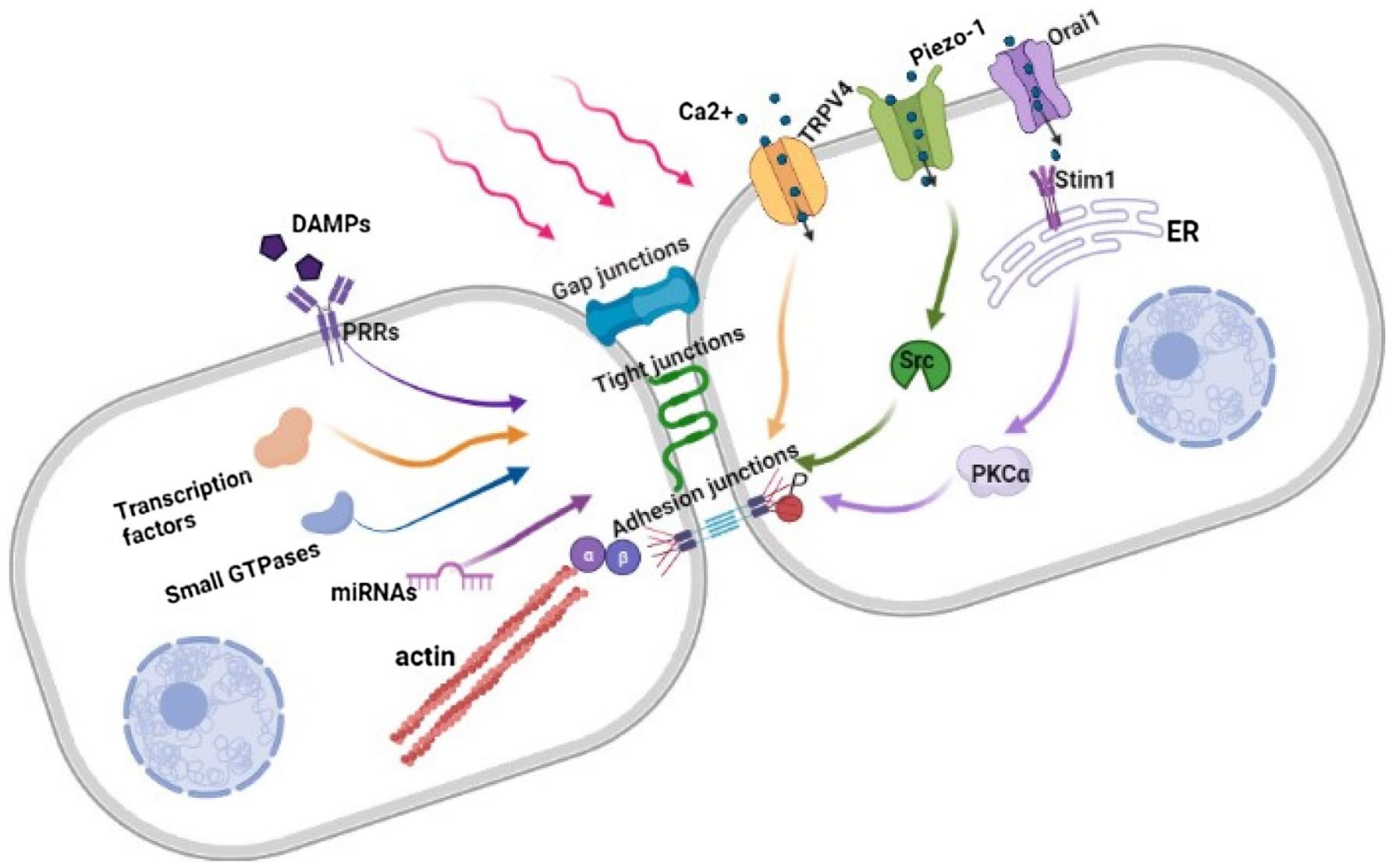
Regulation of Endothelial barrier in response to mechanical force. Under mechanical stimuli, the combination of DAMPs and PRRs, the influx of Ca^2+^ through opened mechanosensitive ion channels and changes in gene expression destruct intercellular structures further leads to cytoskeleton contracting and widening intercellular space. β, β-catenin; α, α-catenin; HMGB1, High-mobility group box 1; DAMPs, Damage-associated molecular patterns; PRRs, Pattern recognition receptors; TRPV4, Transient receptor potential vanilloid 4; Stim1, Stromal-interacting molecule 1; ER, Endoplasmic reticulum; PKCα, Protein kinase C alpha; Src, Proto-oncogene tyrosine-protein kinase.

Endothelial barrier dysfunction is triggered by endothelial sense of mechanical stimuli, which are transformed into biological stimuli and trigger downstream signaling pathways, resulting in cell cytoskeleton remodeling and gene expression changes (Figure 1).

### Cation Channels

Stretch-activated cation channels act as mechanoreceptors and mechanical sensors to mediate mechanical, electrical, and mechanochemical signals (Nilius and Honore, 2012). Stretching activates cation channels by (1) direct tension on the lipid bilayer, (2) transduction of stretch *via* a tethering mechanism from cytoskeletal or extracellular matrix structures, or (3) through releasing or binding of another molecule that in turn activates a channel (Schwingshackl, 2016). The increased pulmonary vascular permeability which caused by excessive lung hyperinflation is associated with activation of cation channels in the vascular endothelium (Lansman et al., 1987; Parker et al., 1998). Previous studies have shown that Ca^2+^ influx or release of stored Ca^2+^ from endoplasmic reticulum activates calmodulin and MLCK. Phosphorylation of MLC increases cytoskeletal tension, intercellular space, and vascular permeability (Wysolmerski and Lagunoff, 1991; Garcia and Schaphorst, 1995; Tiruppathi et al., 2006; Dudek et al., 2010; Parker et al., 2013). Currently, Ca^2+^ channels including TRPs, Stim1/Orai1, and Piezo1 are widely studied.

### Transient Receptor Potential Channels

Transient receptor potential channels (TRPs) are superfamily of ion channels, based on amino acid homologies, the mammalian TRP channel superfamily can be divided into six subfamilies, include TRPV (vanilloid), TRPC (classical or canonical), TRPM (melastatin), TRPP (polycystin), TRPA (ankyrin), and TRPML (mucolipin). TRPs are permeable for Ca^2+^ and play important role in endothelial permeability (Di and Malik, 2010). TRPV4 is abundantly expressed in the lung endothelium and attracts much attention for its influence on mechanical ventilation induced lung endothelial hyperpermeability (Simmons et al., 2019). TRPV4 activation causes Ca^2+^ influx, leading to an increase in the filtration coefficient of lung, but inhibition or knockout of TRPV4 improves pulmonary vascular leakage caused by HTV (Kunert-Keil et al., 2006; Hamanaka et al., 2007; Li et al., 2019; Yu et al., 2020). The protective effect of genetic deficiency or pharmacologic inhibition of TRPV4 may occur due to phosphorylation effect at its serine 824 residues by serum glucocorticoid-regulated kinase (Michalick et al., 2017). Adipose-derived stem cell exosome directly suppresses TRPV4 channel, improves ventilator-induced pulmonary microvascular hyperpermeability as evidenced by lower lung injury score, wet/dry (W/D) weight ratio, and protein concentration in bronchoalveolar lavage fluid (BAL), and increases the expression level of VE-cadherin and β-catenin. Similar to *in vivo* effect, Adipose-derived stem cell exosome decreased intracellular Ca^2+^ concentration and restored endothelial barrier *in vitro* (Yu et al., 2020). For other TRPs, TRPC6 functions as store-operated channels, activation of TRPCs in lung endothelium results in Ca^2+^ influx and subsequent vascular barrier dysfunction and endothelial contraction (Uhlig et al., 2014). TRPC6 was found to be critically involved in lung vascular leakage after stimulation with platelet-activating factor, TRPCs inhibitor or know out TRPC6 obviously attenuated vascular filtration coefficient, and lung edema formation (Samapati et al., 2012). TRPMs are a group of oxidant-activated cation channels, of which TRPM2 is highly expressed in ECs. TRPM2 channels regulate endothelial barrier integrity by facilitating Ca^2+^ influx to ECs, and deletion of TRPM2 in ECs significantly increases the survival rate of endotoxin mice (Mittal et al., 2017). TRPA1 is also known to be activated by oxidative stress in ECs (Simon and Liedtke, 2008). TRPML family has been identified as endolysosomal nonselective cation channels that mediate intra-endosomal calcium release in the endocytic pathway (Villalta and Townsley, 2013). Although TRPC and TRPM families involve in endothelial permeability, we have not found evidence that these channels regulate mechanical ventilation induced lung endothelial permeability (Villalta and Townsley, 2013; Smani et al., 2018; Genova et al., 2020).

### Stim1/Orai1

Stromal-interacting molecule 1 (Stim1) is located in the endoplasmic reticulum and serves as an endoplasmic reticulum Ca^2+^ sensor. Orai1 is the prototypical calcium release-activated Ca^2+^ channel; activated Stim proteins translocate into endoplasmic reticulum plasma membrane junctions and combine directly with Orai1 channels to generate Ca^2+^ entry signals (McNally et al., 2013; Prakriya, 2013). HTV or 18% CS significantly upregulates the expression of Stim1 and Orai1 in pulmonary microvascular ECs, causing an increase in intracellular Ca^2+^ concentration, which activates PKCα to induce increased endothelial permeability. The application of Orai1 inhibitor prevents Ca^2+^ influx and reverses the high permeability of ECs (Song et al., 2018). Stim1-induced Ca^2+^ signaling activates Pyk2 to induce tyrosine phosphorylation of vascular endothelial protein tyrosine phosphatase, which activates Src and subsequently phosphorylates VE-cadherin to increase the endothelial permeability (Soni et al., 2017).

### Piezo-1

Piezo proteins, including Piezo-1 and Piezo-2, are evolutionarily conserved and can respond to various forms of mechanical stimulation including poking, stretching, and shear stress (Geng et al., 2017). Piezo-1, a homotrimeric membrane spanning protein complex that serve as a force sensor, which then responds to mechanical force by regulating the gating of Ca^2+^ (Ge et al., 2015; Zhao et al., 2018). Piezo-1 was first discovered in neuroblastoma cells in mice, and subsequent studies have found that it is also expressed in ECs (Ranade et al., 2014; Cahalan et al., 2015; Ilkan et al., 2017; Liang et al., 2019). Endothelium specifical knock out *Piezo-1* result in embryonic lethality, identifying this channel as a major determinant of vascular development (Li et al., 2014). Piezo-1 is a mechanosensitive Ca^2+^ channel, which can be activated by mechanical force, causing rapid Ca^2+^ influx (Coste et al., 2010). Piezo-1 signaled lung vascular hyperpermeability by promoting internalization and degradation of VE-cadherin, p120-catenin, and β-catenin in ARDS rats undergoing HVT ventilation and LPS-treated ECs exposed to 20% CS (Jiang et al., 2021). But human lung endothelial monolayers depleted of Piezo-1 and exposed to CS show increased permeability (Zhong et al., 2019). Calpain, a calcium-dependent cysteine protease, was identified as a downstream target of Piezo-1 to maintain the endothelial barrier’s stability in response to CS by cleaving Src kinase, which phosphorylates VE-cadherin at Y685 and Y658 (Zhong et al., 2019). Piezo-1 is one of the important mechanosensitive ion channels that participate in endothelial function, more research are need to explore its role in mechanical ventilation induced endothelial permeability.

### Damage-Associated Molecular Patterns

Intracellular molecules released after cell death or subsequent immune cell activation and matrix degradation products are termed as alarmins or damage-associated molecular patterns (DAMPs), which activate pattern recognition receptors to induce multiple signal cascades (Bianchi, 2007). DAMPs include hyaluronan, heat shock proteins, high-mobility box group-1 (HMGB-1), S100 proteins, adenosine 5′-triphosphate, and uric acid. In particular, HMGB-1 has been studied extensively in ECs (Bianchi, 2007; Kuipers et al., 2011). Basic and clinical studies have shown that the expression of DAMPs in BAL of VILI patients was increased, and mechanical ventilation upregulated the expression of pattern recognition receptors in lung (Kuipers et al., 2011). HMGB-1 gene expression is markedly responsive to mechanical ventilation or mechanical stress. HTV of 30ml/kg increased HMGB-1 expression in BAL by five times compared with the control group. HMGB-1 expression was also significantly increased when exposed to excessive and equibiaxial CS *in vitro* (Ogawa et al., 2006; Wolfson et al., 2014; Dong et al., 2015; Feng et al., 2019). Increasing HMGB-1 concentrations induces mitochondrial oxidative damage, resulting in a significant decrease of VE-cadherin and ZO-1 protein that affects intercellular junctions’ integrity and increases endothelial permeability (Dong et al., 2015; Feng et al., 2019) Reducing HMGB-1 release attenuated lung W/D weight ratio and protein concentration in BAL (Wang et al., 2017b; Sun et al., 2019b). For other DAMPs, hyaluronan is a ubiquitous glycosaminoglycan of the extracellular matrix that presents in the endothelial glycocalyx covering the apical surface of ECs. As a key actor of endothelial glycocalyx, hyaluronan plays a central role in endothelial mechanosensing and barrier permeability (Dogne and Flamion, 2020). Different molecular weight of hyaluronan produce distinct biological effects, high-molecular-weight hyaluronan was proved to be an effective treatment strategy for HTV induced lung injury, but low-molecular-weight hyaluronan increased the production of IL-8, which contribute to the development of lung injury (Mascarenhas et al., 2004; Bai et al., 2005). The heat shock proteins (HSPs) are molecular chaperones abundantly present in the cytosol, high peak airway pressure ventilation significantly increased HSP70 expression in lung tissue (Kira et al., 2006). The same as hyaluronan and HSPs, S100 proteins, adenosine 5′-triphosphate, and uric acid also show increased levels during HTV, but the role of these DAMPs in mechanical ventilation induced lung endothelial permeability has not been studied extensively (Kuipers et al., 2011).

### Endothelium Mechanosensitive microRNAs

microRNA (miRNA) is a small non-coding RNA with a length of ~22nt involved in the post-transcriptional regulation of gene expression by binding to 3′UTR region of its mRNA in a non-completely complementary manner (Bartel, 2004; He and Hannon, 2004). miRNAs have been found to be critical modulators of endothelial homeostasis and link to vascular diseases (Fernandez-Hernando and Suarez, 2018). miRNAs in ECs are mechanosensitive that response to mechanical stretch stimulation and exhibited obvious differential expression (Kumar et al., 2014; Zheng et al., 2015). After 1h of mechanical ventilation with a tidal volume of 40ml/kg, the expression of 50 miRNAs increased more than 2-fold, and the expression of 15 miRNAs decreased by more than half (Vaporidi et al., 2012). Among the increased miRNAs, miR-21 promoted the occurrence and development of VILI by targeting the vascular barrier protection factors bone morphogenetic protein receptor type 2 and phosphatase and tensin homolog. The administration of antagomiR-21 significantly ameliorated the indices of high-permeability pulmonary edema caused by mechanical ventilation (Vaporidi et al., 2012). miR-127 has been revealed to be highly expressed in embryos and has been implicated in lung development, placenta formation, and cellular apoptosis (Bhaskaran et al., 2009). In a mature lung, the upregulation of miR-127 expression may contribute to VILI development by increasing pulmonary permeability through NF-κB and p38 MAPK-associated signaling pathways (Li et al., 2017). In contrast to miR-127, miR-135a protects ECs from CS-induced barrier dysfunction, and the protective effects of miR-135a may be related to its inhibition of PH domain leucine-rich repeat protein phosphatase 2 and subsequent activation of PI3K/Akt signaling pathway (Yan et al., 2018). The non-muscle myosin light chain kinase isoform plays a crucial role in agonist-induced pulmonary ECs barrier regulation, and bioinformatic analysis revealed that miR-374 could potentially regulate MYLK gene expression by binding to 3′UTR region (Adyshev et al., 2013). Eighteen percent CS significantly decreases miR-374 expression levels, and functional overexpression of miR-374 attenuates lung MLC subunit activation, which induces the destruction of vascular barrier function (Adyshev et al., 2013). In the miRNA expression profile, ECs specifically express miR-126, which plays a vital role in angiogenesis, endothelial function, and vascular repair (Wang et al., 2008; Chistiakov et al., 2016). In LPS-induced endotoxic mice, miR-126a-3p overexpression decreased blood vessels’ permeability and improved the survival rate of endotoxic mice, but there is no research report on mechanical force effects on miR-126 expression in ECs (Chu et al., 2016). Although the analysis of the role of miRNAs in mechanical force induced endothelial permeability remains in its infancy, the data obtained so far suggest that specifically targeting individual miRNA is a potentially promising therapeutic approach for increased endothelium permeability.

### Transcription Factors

Transcription factors are key cellular components that control gene expression by binding to DNA and other proteins, and their activities determine cells’ function and how they respond to the environment (Spitz and Furlong, 2012). Transcriptional regulation is involved in biomechanical stimuli and controls diverse biological processes (Vaquerizas et al., 2009). Eighteen percent CS significantly increases STAT5 binding to the promoter of nicotinamide phosphoribosyl-transferase (NAMPT), which can exacerbate VILI-mediated inflammation injury *in vivo* (Sun et al., 2014). STAT3 and Nrf2 are critical to 18% CS-induced HMGB-1 expression, which induce increased endothelial permeability in lung ECs (Wolfson et al., 2014; Dong et al., 2015). In pulmonary microvascular ECs, 18% CS reduced the expression of KLF2 by 50% compared with 5% CS, similar to *in vitro* experiments. After 8h of mechanical ventilation, KLF2 expression in the mouse lungs was decreased (Huang et al., 2017). KLF2 confers barrier-protection *via* increasing the expression level of occludin and inhibiting the phosphorylation of MLC, which augments intercellular gaps leading to increased endothelial leakage (Lin et al., 2010). KLF2 also acts as a novel activator of small GTPase Ras-related C3 botulinum toxin substrate 1 by transcriptionally controlling Rap guanine nucleotide exchange factor 3 to maintain vascular integrity (Huang et al., 2017).

### Small GTPases

Small GTPases act as a molecular switch regulated by guanine nucleotide exchange factors (GEFs), GTPase-activating proteins (GAPs), and guanine nucleotide dissociation inhibitors (RhoGDI). The most characterized members, including Rho, Rac, and Cdc42, play a key role in regulating ECs permeability induced by mechanical force (Birukov, 2009). The activation of Rac, Rap1, and Rho induced by CS is amplitude-dependent (Birukov et al., 2003; Birukova et al., 2006). ECs pre- or post-conditioning at low CS magnitude induced Rap1 activation, which promotes the resealing of cell junctions disrupted by high CS magnitude. The protective effect was abolished by pharmacological or molecular inhibition of Rap1 activity (Ke et al., 2019). As a Rap1-binding protein, Krev interaction trapped-1 knockdown exacerbated Rho activation and ECs barrier disruption induced by 18% CS (Meliton et al., 2015). Five percent CS causes Rac activation and promotes the recovery of endothelium monolayer integrity after thrombin stimulation, but 18% CS increased thrombin-induced Rho activity (Birukova et al., 2006). HTV increased RhoA/Rock protein expression (Dai et al., 2019a). The activation of RhoA induces the phosphorylation of MLC and contraction of ECs, resulting in gap formation and increased endothelial permeability (Liu et al., 2013). Rock inhibitor could attenuate CS-induced lung endothelial hyperpermeability and the phosphorylation of myosin phosphatase target subunit 1 *in vivo* and *in vitro* (Wang et al., 2019). 1-palmitoyl-2-arachidonoyl-snglycero-3-phosphatidyl choline showed a protective role of stretch-induced barrier-disruptive *via* triggering Rac activation (Birukova et al., 2011).

## Conclusion

Inappropriate mechanical ventilation is confirmed to disrupt pulmonary endothelial barrier. In this review, we discussed the endothelial barrier dysfunction caused by mechanical force. As discussed above, the maintenance of endothelial barrier depends on the stability of various intercellular structures, the open and close of mechanosensitive ion channels and changes in gene expression. The mechanisms involved in hyperpermeability of endothelial are not fully revealed, and most studies focused on individual component. We suppose that a more holistic understanding of underlying signaling mechanisms in mechanical force that induce endothelial barrier dysfunction could lead to the discovery of new therapeutic targets during VILI.

## Author Contributions

YL wrote the manuscript. YH obtained funding and reviewed the manuscript. All authors contributed to the article and approved the submitted version.

## Funding

This work was supported by the National Natural Science Foundation of China (81870069), the Natural Science Foundation of Guangdong Province, China (2017A030313781), and the Science and Technology Program of Guangzhou, China (202102010366).

## Conflict of Interest

The authors declare that the research was conducted in the absence of any commercial or financial relationships that could be construed as a potential conflict of interest.

## Publisher’s Note

All claims expressed in this article are solely those of the authors and do not necessarily represent those of their affiliated organizations, or those of the publisher, the editors and the reviewers. Any product that may be evaluated in this article, or claim that may be made by its manufacturer, is not guaranteed or endorsed by the publisher.
